# Single-step isolation of carbon nanotubes with narrow-band light emission characteristics

**DOI:** 10.1038/s41598-018-37675-4

**Published:** 2019-01-24

**Authors:** Edyta Turek, Tomohiro Shiraki, Tomonari Shiraishi, Tamehito Shiga, Tsuyohiko Fujigaya, Dawid Janas

**Affiliations:** 1 Department of Chemistry, Silesian University of Technology, B. Krzywoustego 4, 44-100 Gliwice, Poland; 2 Department of Applied Chemistry, Graduate School of Engineering, Kyushu University, 744 Motooka, Nishi-ku, Fukuoka 819-0395 Japan

## Abstract

Lack of necessary degree of control over carbon nanotube (CNT) structure has remained a major impediment factor for making significant advances using this material since it was discovered. Recently, a wide range of promising sorting methods emerged as an antidote to this problem, all of which unfortunately have a multistep nature. Here we report that desired type of CNTs can be targeted and isolated in a single step using modified aqueous two-phase extraction. We achieve this by introducing hydration modulating agents, which are able to tune the arrangement of surfactants on their surface, and hence make selected CNTs highly hydrophobic or hydrophilic. This allows for separation of minor chiral species from the CNT mixture with up to 99.7 ± 0.02% selectivity without the need to carry out any unnecessary iterations. Interestingly, our strategy is also able to enrich the optical emission from CNTs under selected conditions.

## Introduction

Single-walled carbon nanotubes (SWCNTs) of defined chiral index are a very promising material for many optical applications ranging from optical sensors^1^, photothermal therapy^2^ to light emitters at telecom wavelengths^3^. Unfortunately, despite many efforts, straightforward synthesis of SWCNTs of particular chirality is still at its infancy and only a selected number of SWCNTs can be obtained this way^4^. Moreover, the material comes in very small amounts and it is always just enriched with certain SWCNT type. An arsenal of post-synthesis sorting methods has been established to tackle this problem^5–9^. Aqueous two-phase extraction (ATPE), which was developed for separation of cell particles in 1970^10^, has recently proven to be a very rapid and convenient method of separation of SWCNT mixtures^11–14^. In this approach, SWCNTs partition between two phases of the dextran – polyethylene glycol (PEG) system. Stepwise change of surfactant concentration enables one to separate particular CNT types with single-chirality resolution. Unfortunately, to isolate CNTs of a given chirality one has execute a number of iterations despite the fact that often significant amount of material is lost at the interface with each partitioning step^15^.

**Figure 1:**
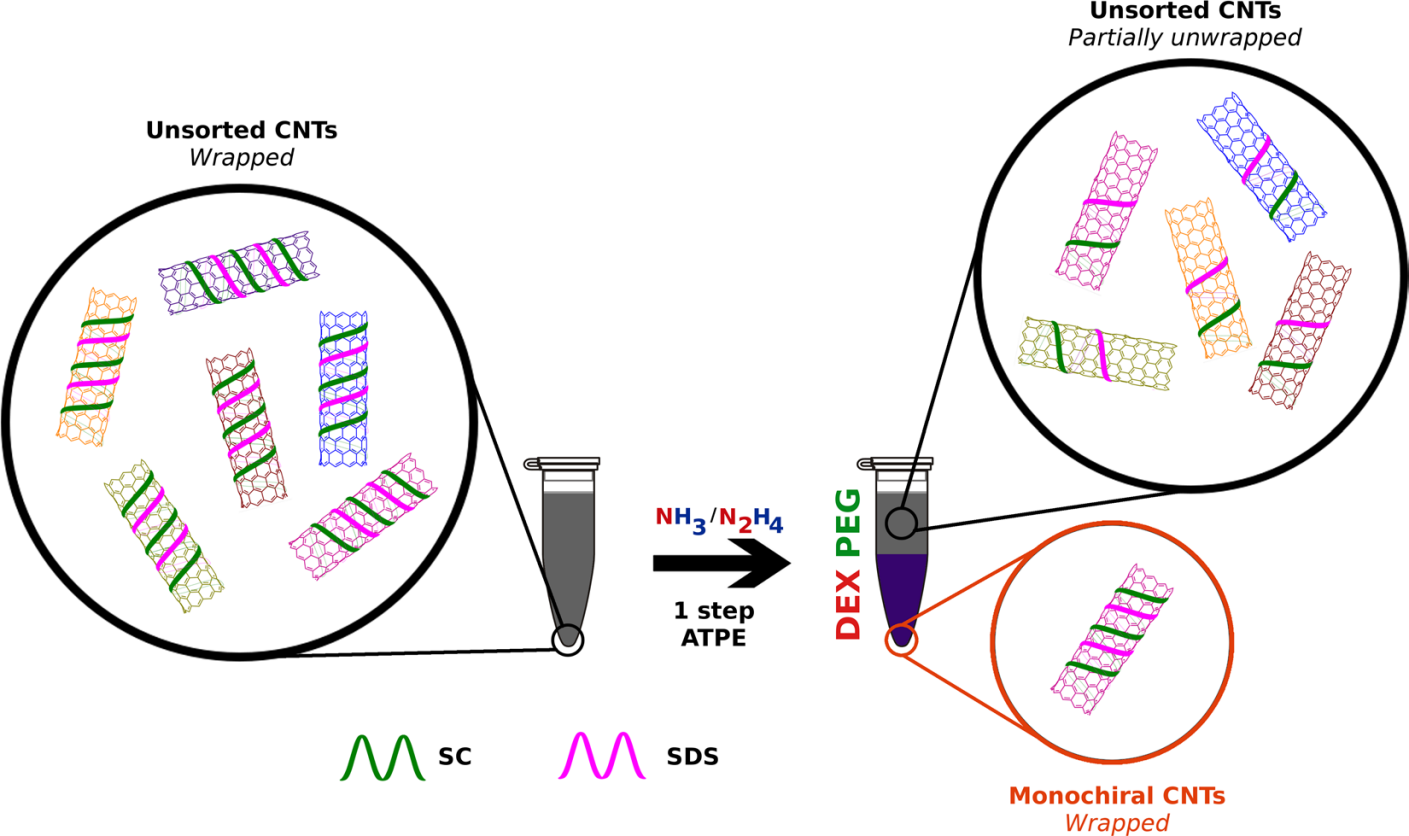
Single-step sorting of CNTs by modified aqueous two-phase extraction (ATPE).

Herein, for the first time, we demonstrate a new approach, which enables separation of nearly monochiral semiconducting SWCNT fractions in just a single step. The key was to introduce hydration modulating agents, which influenced the arrangement of surfactants on the surface of SWCNTs (Fig. 1). That particular order has got a predominant effect on the shape of the hydration sphere around individual SWCNTs, which in turn determines the solubility of CNTs of a particular type in one of the two phases. We show that by careful adjustment of type and amount of surfactant and hydration modulator it is indeed possible to target CNTs of a selected diameter and extract it from the mixture. Interestingly, even minor SWCNT types can be separated this way reaching material of nearly homogeneous light emission characteristics in one go. The presented approach can be useful for separation of all kinds of molecules by ATPE – not just SWCNTs.

## Experimental

For this work, we have used DEX (dextran), PEG (poly(ethylene glycol)), SC (sodium cholate), SDS (sodium dodecyl sulfate) and H_2_O or D_2_O (where indicated). When D_2_O was employed, all the chemical compounds for ATPE separation were also dissolved in D_2_O prior to the experiment. Aqueous solutions of NH_3_ or N_2_H_4_ were used as hydration modulating agents. Appropriate amounts of all the ATPE ingredients were combined in an Eppendorf tube with H_2_O or D_2_O dispersion of HiPco SWCNTs dispersed previously by sonication using 2% SC. After vigorous shaking to mix all the components, the tubes were gently centrifuged to speed up the separation of the two phases.

## Results

### Separation of (6,5) and (8,3) rich CNT fractions

First, we would like to comment on the selection of the parent material for the study. HiPco CNTs were chosen as for a separation approach to be successful it has to be effective in discrimination between a large number of CNTs. One of the common weak points of many studies on CNT separation is using materials such as (6,5) rich CNTs produced by CoMoCAT system with a dominant chirality^16^.

**Figure 2:**
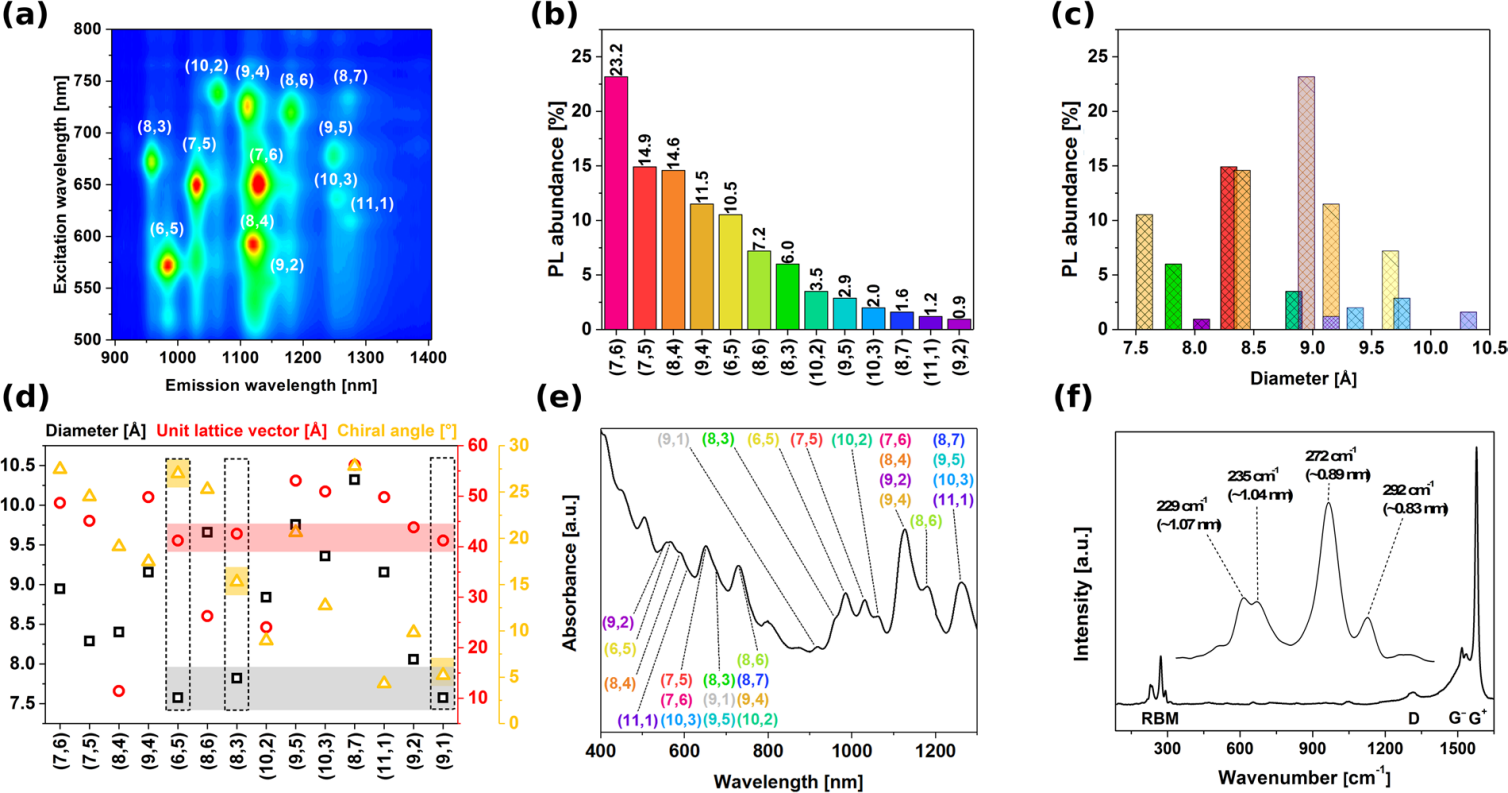
Characterization of the starting CNT material (HiPco): (**a**) 2D PL map, (**b**) Abundance expressed by PL intensity, (**c**) corresponding diameter distribution, (**d**) analysis of parameters of constituting CNTs, (**e**) absorbance spectrum and (**f**) Raman spectrum (magnification of the RBM area in the inset).

As compared with HiPco (Fig. 2), which we found to contain at least 13 different CNT chiralities of semiconducting type (metallic are not visible by PL), the (6,5) rich CoMoCAT is composed of no less than 6 chiralities (Fig. S2). One actually has to keep in mind, when using it as the source of (6,5) CNTs for the experiment, that the sorting step is necessary for precise correlation of the studied property with chiral angle. Unfortunately, this is often neglected. Leaving the digression aside, PL measurements showed that the strongest signals come from (7,6) CNTs, which are also the most abundant fraction as measured by absorbance spectroscopy. Observed diameter distribution gauged by Raman spectroscopy and recalculated from chiral indices^17^, centred at 0.9 nm, falls within the values given by the manufacturer for this sample (0.8–1.2 nm). Figure 2d depicts the characteristics of each detected semiconducting CNT chirality in the sample based on the tabular data provided by Kataura *et al*.^17^ *(description of isolated CNTs indicated with dashed rectangles is given in the later part of the manuscript)*. Due to the resonant nature of Raman spectroscopy, just a selected subset excited at 633 nm was detected. The G mode split into G− and G+ sub-bands as expected for high purity single-walled CNT material.

**Figure 3:**
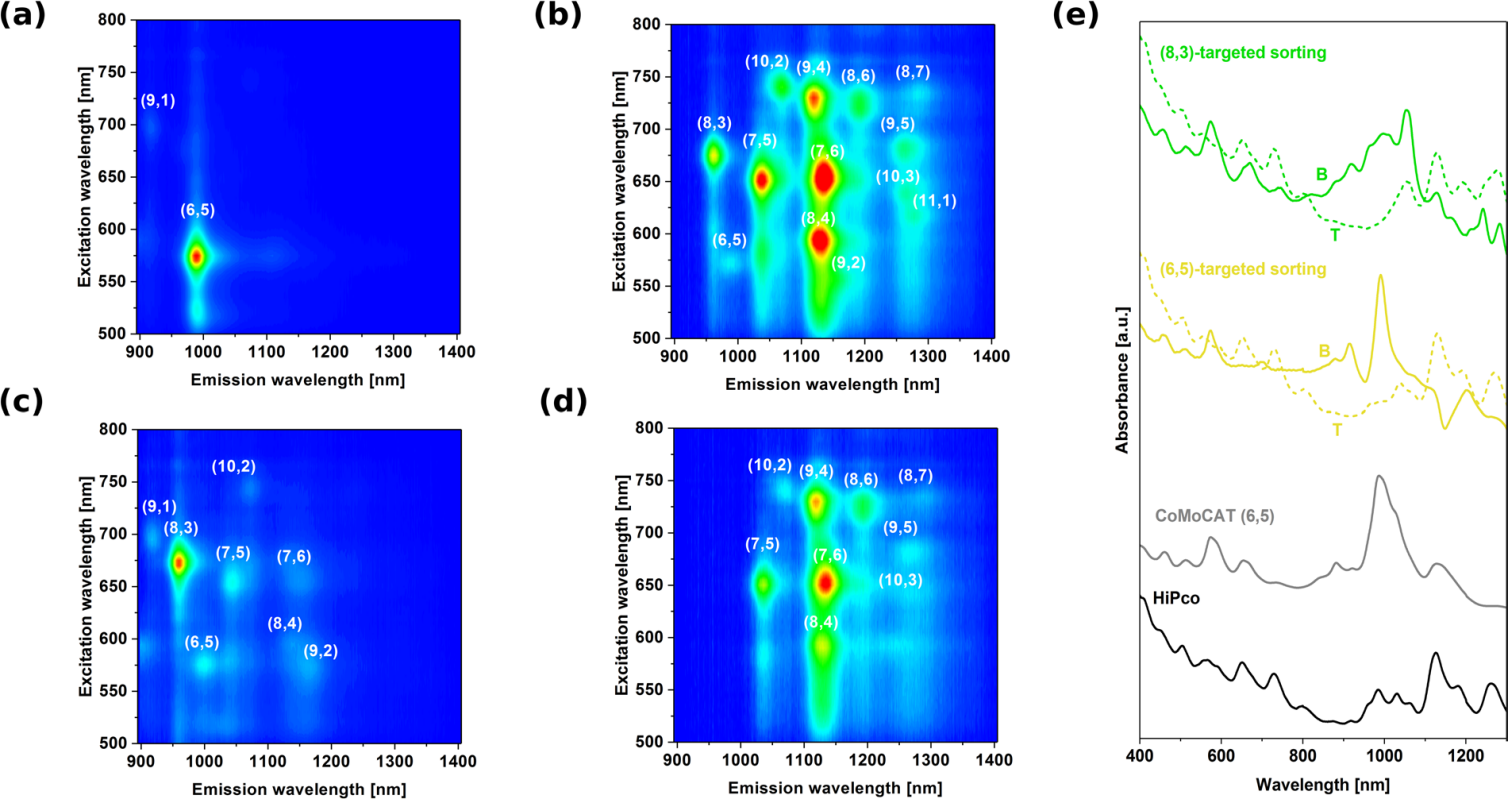
Characterization of the sorted CNT materials by one-step ATPE: (**a**) (6,5)-rich CNT fraction (75 μL CNT + 20 μL NH_3_, bottom phase), (**b**) corresponding top phase, (**c**) (8,3)-rich CNT fraction (150 μL CNT + 200 μL N_2_H_4_, bottom phase) and (**d**) corresponding top phase. (**e**) Absorbance spectra of parent material (HiPco), CoMoCAT (6,5)-rich reference and all four phases described above (targeted for (6,5) and (8,3) CNT extraction).

Introduction of additional components into the biphasic system can have a strong influence on the affinity of particular type of CNTs towards one of the two phases^4^. One approach involves addition of chaotropic (*e*.*g*. NaSCN)^11^ or kosmotropic salts (*e*.*g*. NaCl)^18^, which shift CNTs from the top phase to the bottom or conversely, respectively. The underlying reason for the observed phenomenon is the fact that these agents affect the CNT-surfactant hydration layer, which is a key factor for differentiation of CNTs by ATPE. Furthermore, redox molecules, which cause electron transfer from or to the CNTs can influence the end result of ATPE of CNTs. Gui *et al*. showed that NaBH_4_ reductant or NaClO oxidant can also change the shape of the surfactant coating on the CNTs and hence the hydration layer^12^. We have found out that ammonia and its “dimer” hydrazine not only makes favourable shifts of CNTs between the two phases, but the redistribution is sensitive to particular diameters (Fig. 3). (6,5) CNTs, which are a minor fraction of HiPco material, were isolated in just a single step with 99.7 ± 0.02% yield among the whole semiconducting CNT population using NH_3_ as an extra ATPE ingredient (Figs 3a and S7). The only remaining other semiconducting CNT chirality in the bottom phase was (9,1), which is of exactly the same diameter as (6,5), so we suspect that the separation is diameter selective. In fact, (6,5) CNTs are the smallest diameter CNTs among all the other HiPco species (Fig. 2d). The absorbance spectra closely match that of (6,5) rich CoMoCAT material, but the peaks are more defined because of much higher purity. The top phase (Fig. 3b) shows near exclusion of (6,5) CNTs, which account for only 0.6% of light emission in this fraction (Fig. S8). It is a remarkable result achieved with just one step of separation. Interestingly, SC was used to make the parent CNT dispersions. Most commonly SC-wrapped CNTs separate according to the electrical character^6,19^ or give fractions of particular, but rather broad diameter distribution^20^.

As postulated by Arnold *et al*. bile salt structure such as that of SC is much more rigid than SDS or SDBS^6^, therefore it cannot form a hemispherical micelle, but instead the molecules order around a CNT with their less polar sides faced towards the CNT^21^. Such arrangement is inherently more sensitive due to the much lower stability of that system.

**Figure 4:**
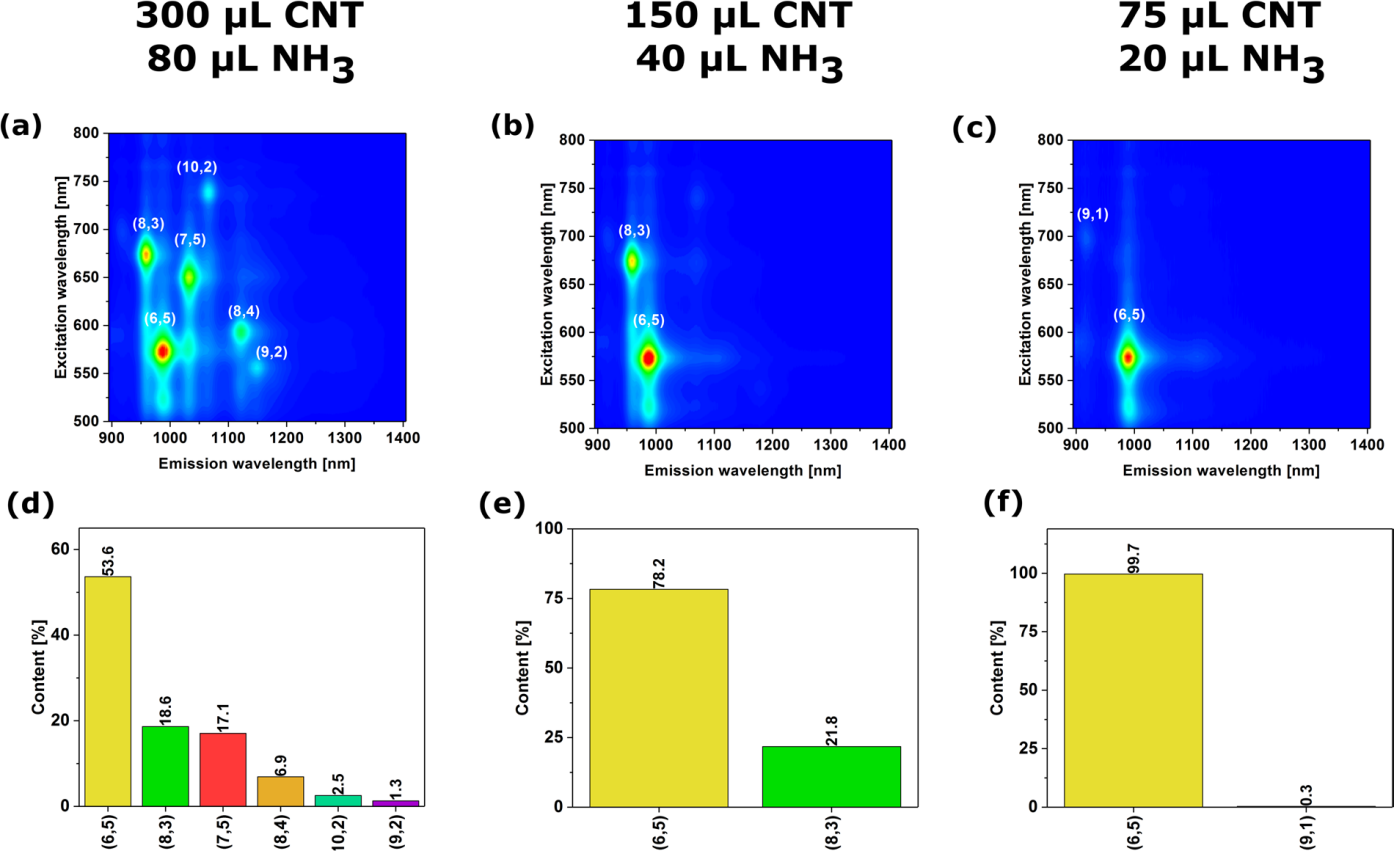
The influence of the amount of CNTs and hydration modulator on the end result of ATPE separation (top – 2D PL maps, bottom – corresponding absorbance spectra).

The results show that not only the relative amount of ammonia to CNTs is relevant, but the composition of the whole ATPE system has to be tuned to extract a particular CNT type (Figs 4 and S3–S8). In general, higher CNT loadings (ca. 300 μL) lead to poor separation (about 6 chiralities in the resulting sample). Reducing the amount of injected CNTs by half affords CNT mixture of just two semiconducting chiralities as measured by PL, but the content of targeted (6,5) CNTs is only at the level of 75%. Only when the amount of introduced CNTs is reduced to 75 μL (for the selected total volume of 1.53 mL of the ATPE system) and the ammonia amount is low (20 μL), then the separation is successful.

**Figure 5:**
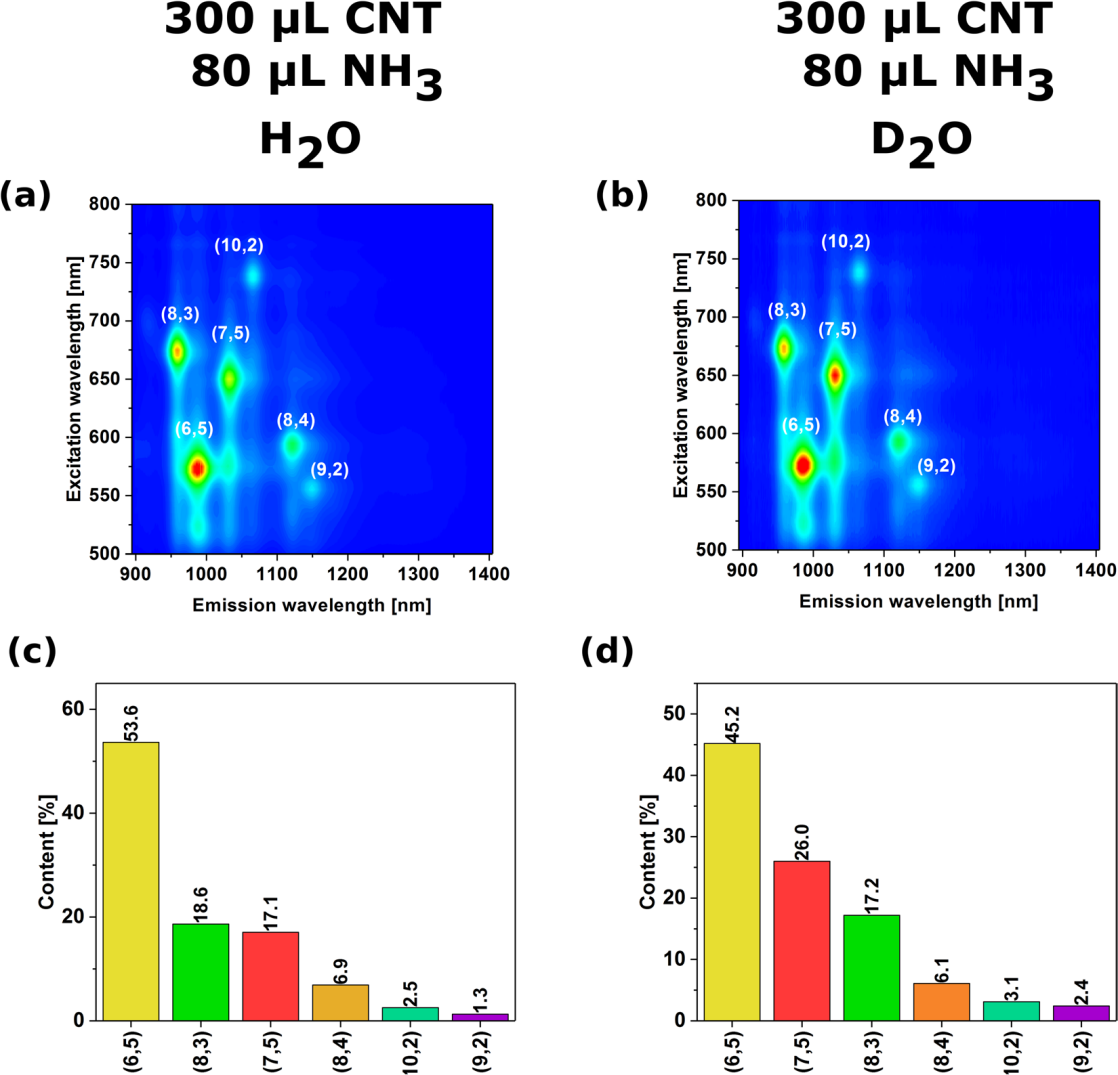
The influence of the type of solvent on the end result of ATPE separation (top – 2D PL maps, bottom – corresponding absorbance spectra).

It is very interesting to note that even the isotope effect can put the ATPE out of tune. When we prepared the samples for PL analysis in D_2_O the results were different that when H_2_O was the solvent (Figs 5 and S9–12) – the mixtures were more polydisperse or the content of (6,5) CNTs was less dominant. D_2_O is over 11% more dense than H_2_O, much less dissociated and has a slightly higher dipole moment^22^, all of which may affect the sorting behaviour.

Furthermore, separation aimed at isolation of (8,3) chirality was possible (using N_2_H_4_ instead of NH_3_ and increasing the ratio of hydration modulator to CNTs above unity), but the yield of the received material was not so high as in the case of (6,5) CNTs (Fig. 3c,d). 83 ± 3% of the emission signal came from this type of CNTs (Figs 3c and S15). Nevertheless, it is intriguing to see that the ATPE preference can be steered towards particular CNT diameter distribution and enable their isolation (with minor contamination) in just a single step. This means that the system is in theory capable of isolation of monochiral semiconducting CNT fractions provided that diameters of constituting CNTs in the parent mixture are sufficiently separated from each other beyond a certain threshold. When we compare 83% of the PL coming from (8,3) CNTs observed in this study with commercially available (6,5)-rich material CoMoCAT, for which the PL purity is just a the level of 56% (Fig. S2), the results presented herein become very encouraging. There is large number of studies based on as-is (6,5)-rich CoMoCAT^23–25^ obtained from commercial sources because access to different CNT chiralities is restricted. By using the simple one step ATPE approach presented herein and widely available HiPco material the inherent role of chiral index in CNTs could be studied in greater detail already. Moreover, the 2D PL map of the corresponding top phase shows complete exclusion of (8,3) species from it (Fig. 3d). Injection of higher content of CNTs than the standard 75 μL did not lead to successful separation again (Figs S13 and S14).

### Emergence of a new emission peak

**Figure 6:**
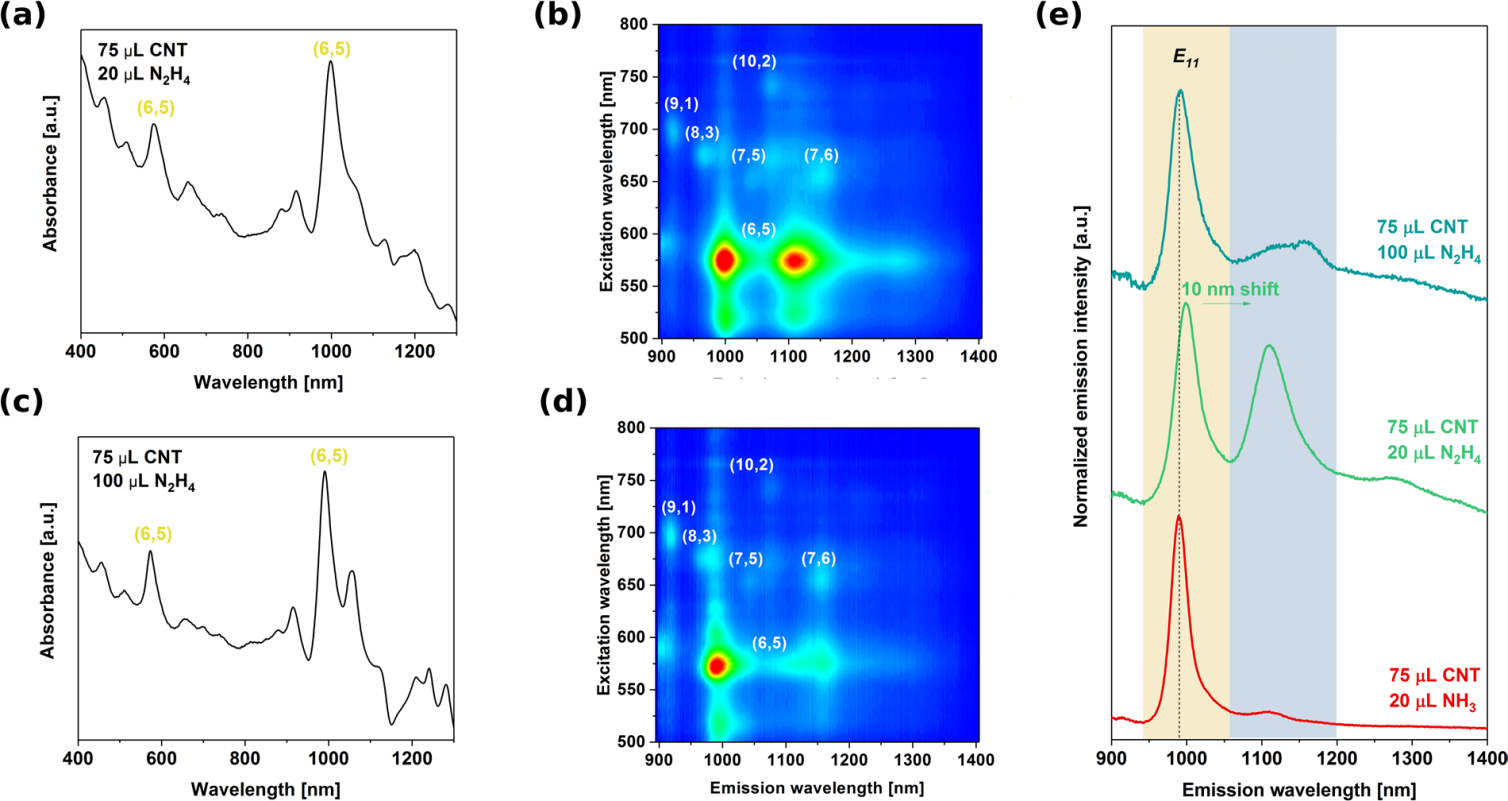
Evolution of new PL modes (**a**,**b**) corresponding absorbance spectra, (**c**,**d**) 2D PL maps and (**e**) PL profiles at 575 nm excitation wavelength (red curve is a reference PL profile from near monochiral (6,5) CNTs obtained in the previous section).

Finally, we would like to report on the unexpected emergence of a new and strong peak at 1100 nm, which is red-shifted from the E_11_ PL centered at 990 nm from non-functionalized CNTs (Fig. 6). Here two possibilities might be considered as the origin of the new emission. Such red-shifted emission called E_11_^*^ has been reported when CNTs were functionalized by slight oxidation^26,27^, alkylation^28,29^ or arylation *via* diazonium chemistry^30,31^. In such case, dramatic improvement in the luminescence yield was observed. Here, it appears that optically allowed states below the energy levels of dark excitions can be created by simple electron transfer process from the hydrazine molecule to the CNTs. The amount of hydrazine required to cause this effect is orders of magnitude larger than by using the functionalization approach, wherein the new emission peaks emerge only at the nanomolar level (1:1000 carbon atoms is functionalized or less) and further addition causes quenching of PL altogether.

**Figure 7:**
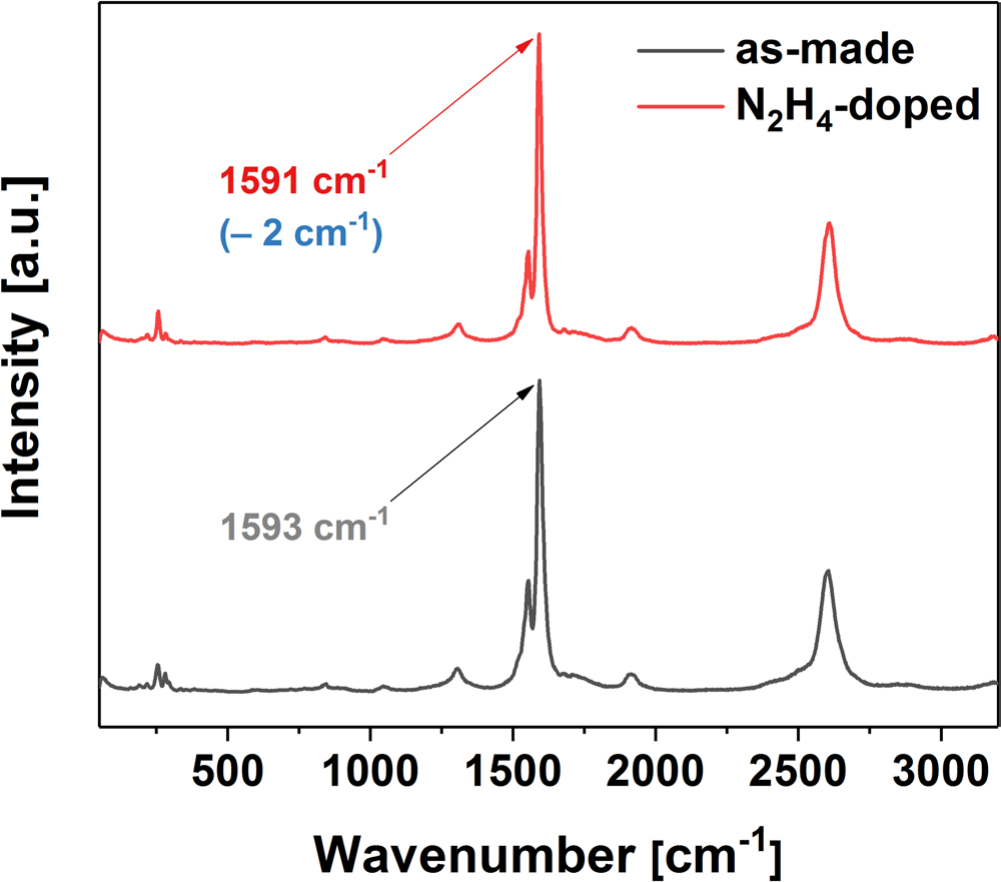
Raman spectra of parent CNTs (as-made and doped with hydrazine).

If CNTs at the starting concentration of 1 mg/mL (some of which were discarded after centrifugation) were to partition equally by weight between the top and the bottom phase (without any material lost at the interface), the weight ratio of the CNTs to the hydrazine (assuming uniform concentration of hydrazine throughout the whole ATPE system) would be on the order of unity (~7.50 for 75/20 combination and ~1.50 for 75/100 combination). Increase of hydrazine concentration fivefold suppresses the new signal. In contrast with the approach of defect engineering, exceeding the threshold does not quench the E_11_ emission signature, but just the E_11_^*^-like mode. The new emission feature is evident only in the bottom phase (Figs S17–19). Due to the very pristine nature of the starting material we can exclude the reducing action of hydrazine, which we confirmed by Raman spectroscopy, wherein no change to the I_D_/I_G_ ratio was observed. The G+ peak however was slightly red-shifted, which indicates n-doping of the material (Fig. 7)^32^.

Nevertheless, so far only these two combinations of parameters led to the evolution of new PL feature, which means that this new mode requires very strict conditions to emerge (even when only H_2_O was swapped for D_2_O with all other conditions kept intact, the apparent E_11_* did not appear). Furthermore, Matsunaga *et al*. indicated that p-doped CNTs may show emission peaks resembling E11*, but their intensity was much lower than those of observed by us. The effect was explained by the formation of trions at room temperature^33^. Hydrazine, which we used, is a known n-doping agent of nanocarbon materials, so we can not exclude this phenomenon as the underlying reason for emergence of a new spectral feature herein.

## Conclusions

In summary, we have demonstrated that sorting of CNTs can be accomplished in a single step reaching nearly homogeneous light emission characteristics. For the isolation to be successful, an ATPE sweet spot has to be reached because even the slightest deviation from the selected conditions leads to ineffective differentiation. However, high sensitivity enables tuning of the system towards extraction of different CNT diameter distribution, which, under certain conditions, corresponds to nearly monochiral fractions. Moreover, by exposing CNTs to hydrazine under particular conditions we were able to create new optically allowed states. Finally, since ATPE is not just a method to process CNTs, but constitutes an important tool for molecular biology, we believe that our findings can be helpful to a much wider audience.

## Supplementary information

Supplementary Information file

## Data Availability

Available from the corresponding author (D.J.).
