# Supplementary material for: Single-step isolation of carbon nanotubes with narrow-band light emission characteristics
Source: Sci Rep. 2019 Jan 24;9:535. doi: 10.1038/s41598-018-37675-4 (PMC6345979; doi:10.1038/s41598-018-37675-4)
Supplement: Supplementary file 1 — Supplementary Information file [file 41598_2018_37675_MOESM1_ESM.docx]

**Electronic supplementary information**

for

**Single-step isolation of carbon nanotubes with narrow-band light emission characteristics**

by

Edyta Turek^a^, Tomohiro Shiraki^b^, Tomonari Shiraishi^b^, Tamehito Shiga^b^, Tsuyohiko Fujigaya^b^, Dawid Janas^a,^[[1]](#footnote-1)^^

*^a^* *Department of Chemistry, Silesian University of Technology, B. Krzywoustego 4, 44-100 Gliwice, Poland*

*^b^* *Department of Applied Chemistry, Graduate School of Engineering, Kyushu University, 744 Motooka, Nishi-ku, Fukuoka 819-0395, Japan*

Corresponding author:
Tel.: + 48 32 2372958

E-mail address: Dawid.Janas@polsl.pl (Dawid Janas)

1. **Experimental**
   1. Dispersion of SWCNTs

Single-walled carbon nanotubes (SWCNTs) made by the HiPco method were obtained from Nanointegris (0.8 – 1.2 nm, SKU 1603, HS 28030). The CNTs were dispersed in H_2_O (1 mg/mL concentration) or D_2_O (where indicated) by sonication for 2 hours (Hielscher UP50H) in the presence of SC (2%). During sonication, the material was kept in an ice-bath to improve the quality of the dispersion. Afterwards, the dispersion was centrifuged at 11,000 rpm (Eppendorf 5804R centrifuge) for 2 h to remove the non-individualized CNTs. Upper 80% of the supernatant was decanted and used for the study. CoMoCAT (6,5) enriched CNTs were purchased from SouthWest Nanotechnologies and used as reference.

- 1. Separation of SWCNTs

One-step separation by ATPE was carried out by modifying an approach published by Gui *et al.* ^12^ CNT dispersion, Dextran (20%, aq.), PEG (50%, aq.), SC (10%, aq.), SDS (10%, aq.), hydration modulator and H_2_O were combined in an Eppendorf tube. For the HiPco dispersions in D_2_O, all the added chemicals were also dissolved in D_2_O. The mixture was shaken until all the components were homogeneously dispersed. Two phases would spontaneously separate over time, but, to speed up the process, the mixture was centrifuged for 3 minutes at 2,000 rpm to make the PEG and DEX emerge.

- 1. Characterization

2D Photoluminescence maps were acquired using Horiba Jobin Yvon spectrofluorometer (FluorologR-3 with FluorEssence). Integration time was varied to detect all the CNT species present in the sample. In particular, the spectra acquired in H_2_O had longer integration times than those in D_2_O due to the fact that water reabsorbs the emitted radiation from the samples.
To enable comparison between the samples, the maps are normalized to the strongest CNT emission peak recorded in a particular map. Composition of the samples was calculated by integrating the volume of the respective peaks from the 2D PL maps. Since not all CNTs emit with the same quantum yield, the quoted content values should be understood in terms of optical purity of the samples. An example how this was done is given below (Fig. S1).


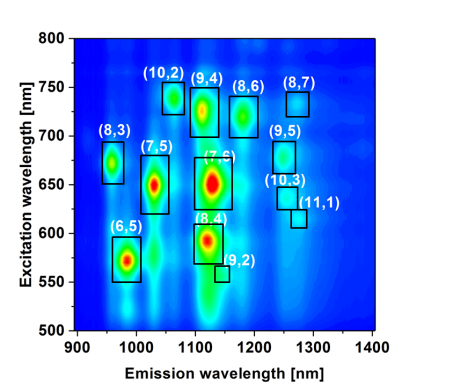


**Figure S1** Evaluation of abundance by integration of respective emission peaks in 2D PL maps.

Absorbance spectra were obtained from 400 to 1300 nm using V-670 (JASCO) spectroscope.

Raman spectra were registered by a RAMANtouch spectrometer (Nanophoton Corporation).
The analyzed samples were in the liquid form.

1. **Results and discussion**
   1. (6,5) CoMoCAT CNTs


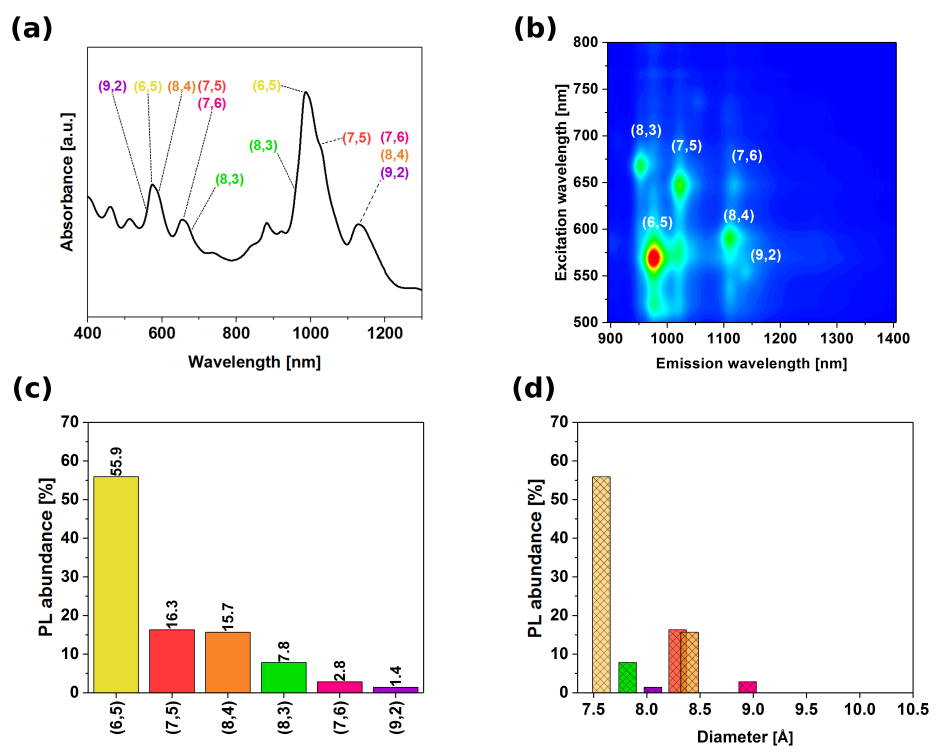


**Figure S2** Characterization of CoMoCAT material: (a) absorbance spectra, (b) 2D PL map, (c) Abundance expressed by PL intensity and (d) corresponding diameter distribution.

CoMoCAT CNT material is composed of at least 6 different semiconducting chiralities, in which (6,5) is the dominant one as proven by absorbance spectroscopy and PL measurements (Figure S2). It is important to mention, that purification of this material by sorting is critical for optical applications as (6,5) CNTs account only for 55.9% of measured light emission from the sample.

- 1. (6,5) CNTs extraction experiments


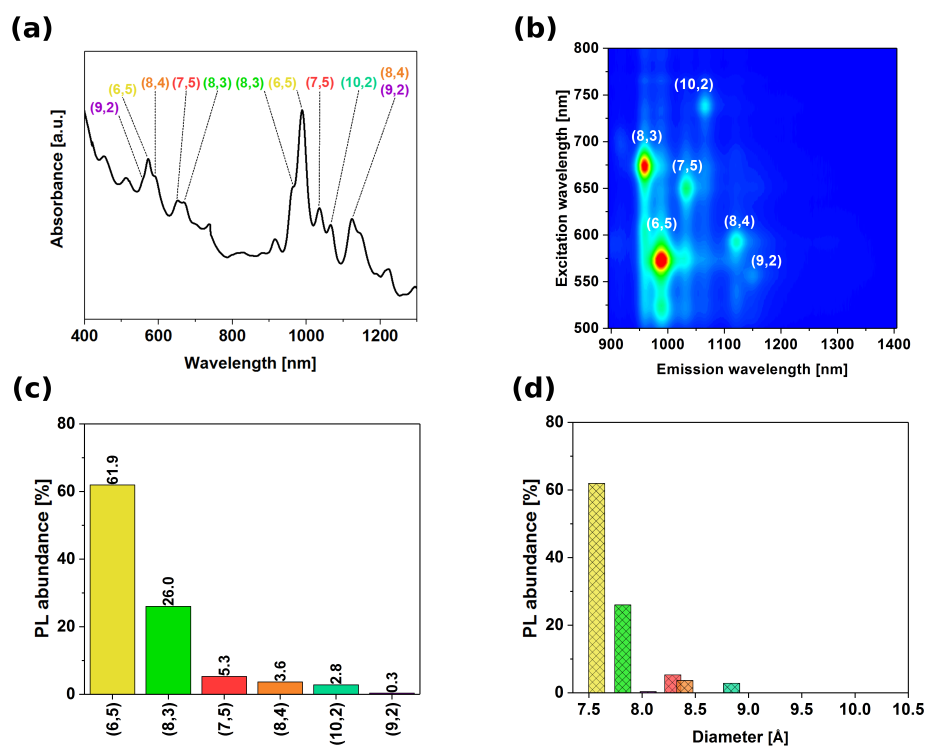


**Figure S3** Characterization of the bottom ATPE phase after combination of 300 µL CNT + 400 µL NH_3_ (a) absorbance spectrum, (b) 2D PL map, (c) Abundance expressed by PL intensity and (d) corresponding diameter distribution.

Table 1. ATPE parameters

| **Compound** | **Aq. concentration [%]** | **Volume [µL]** |
| --- | --- | --- |
| DEX | 20 | 450 |
| PEG | 50 | 180 |
| SC | 10 | 120 |
| SDS | 10 | 60 |
| CNTs | 2% SC, 1 mg/mL | 300 |
| NH_3_ (aq.) | 25 | 400 |
| H_2_O | 100 | 20 |


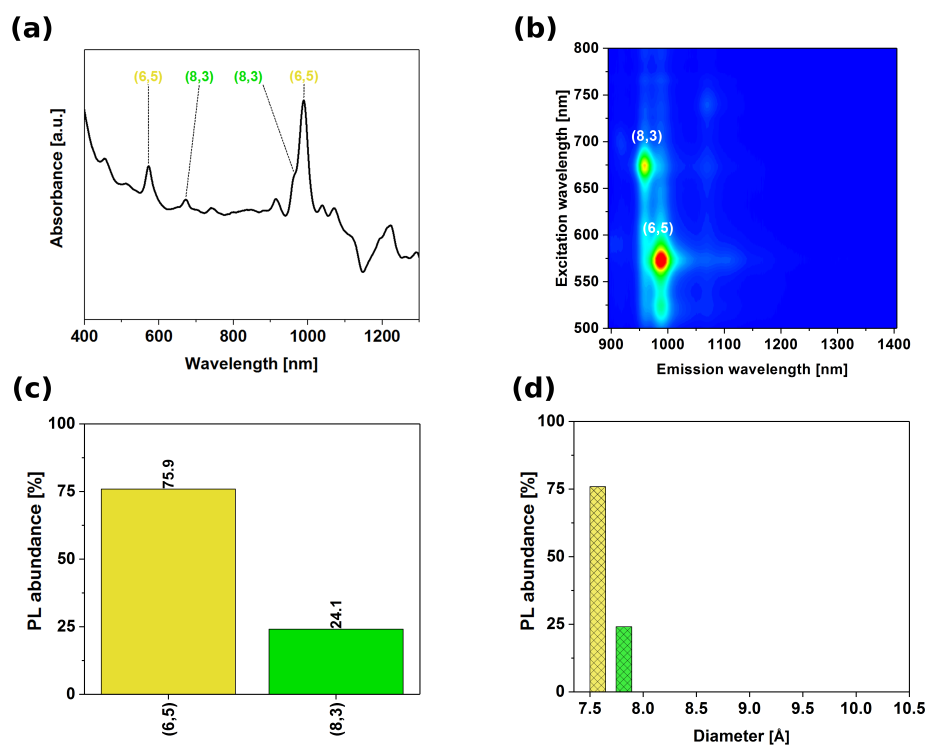


**Figure S4** Characterization of the bottom ATPE phase after combination of 150 µL CNT + 200 µL NH_3_ (a) absorbance spectrum, (b) 2D PL map, (c) Abundance expressed by PL intensity and (d) corresponding diameter distribution.

Table 2. ATPE parameters

| **Compound** | **Aq. concentration [%]** | **Volume [µL]** |
| --- | --- | --- |
| DEX | 20 | 450 |
| PEG | 50 | 180 |
| SC | 10 | 120 |
| SDS | 10 | 60 |
| CNTs | 2% SC, 1 mg/mL | 150 |
| NH_3_ (aq.) | 25 | 200 |
| H_2_O | 100 | 370 |


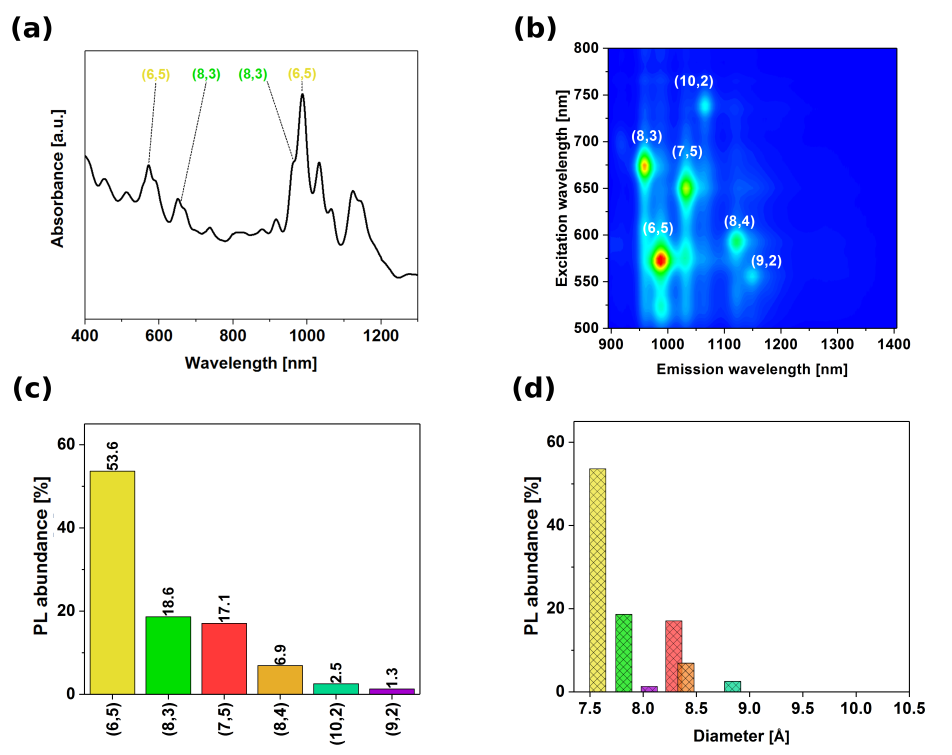


**Figure S5** Characterization of the bottom ATPE phase after combination of 300 µL CNT + 80 µL NH_3_ (a) absorbance spectrum, (b) 2D PL map, (c) Abundance expressed by PL intensity and (d) corresponding diameter distribution.

Table 3. ATPE parameters

| **Compound** | **Aq. concentration [%]** | **Volume [µL]** |
| --- | --- | --- |
| DEX | 20 | 450 |
| PEG | 50 | 180 |
| SC | 10 | 120 |
| SDS | 10 | 60 |
| CNTs | 2% SC, 1 mg/mL | 300 |
| NH_3_ (aq.) | 25 | 80 |
| H_2_O | 100 | 340 |


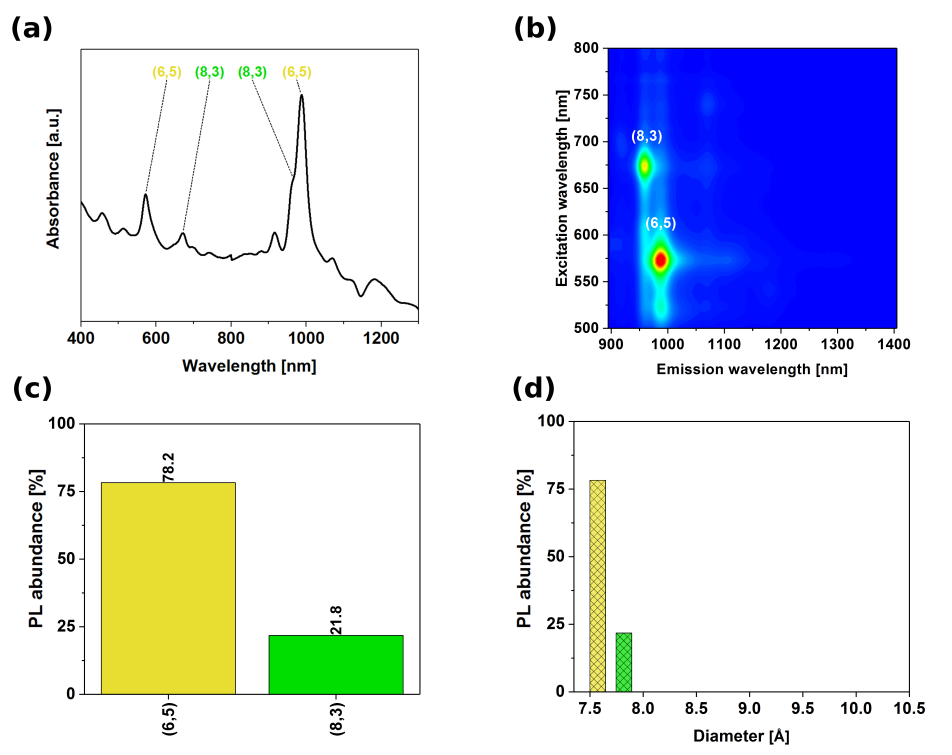


**Figure S6** Characterization of the bottom ATPE phase after combination of 150 µL CNT + 40 µL NH_3_ (a) absorbance spectrum, (b) 2D PL map, (c) Abundance expressed by PL intensity and (d) corresponding diameter distribution.

Table 4. ATPE parameters

| **Compound** | **Aq. concentration [%]** | **Volume [µL]** |
| --- | --- | --- |
| DEX | 20 | 450 |
| PEG | 50 | 180 |
| SC | 10 | 120 |
| SDS | 10 | 60 |
| CNTs | 2% SC, 1 mg/mL | 150 |
| NH_3_ (aq.) | 25 | 40 |
| H_2_O | 100 | 530 |


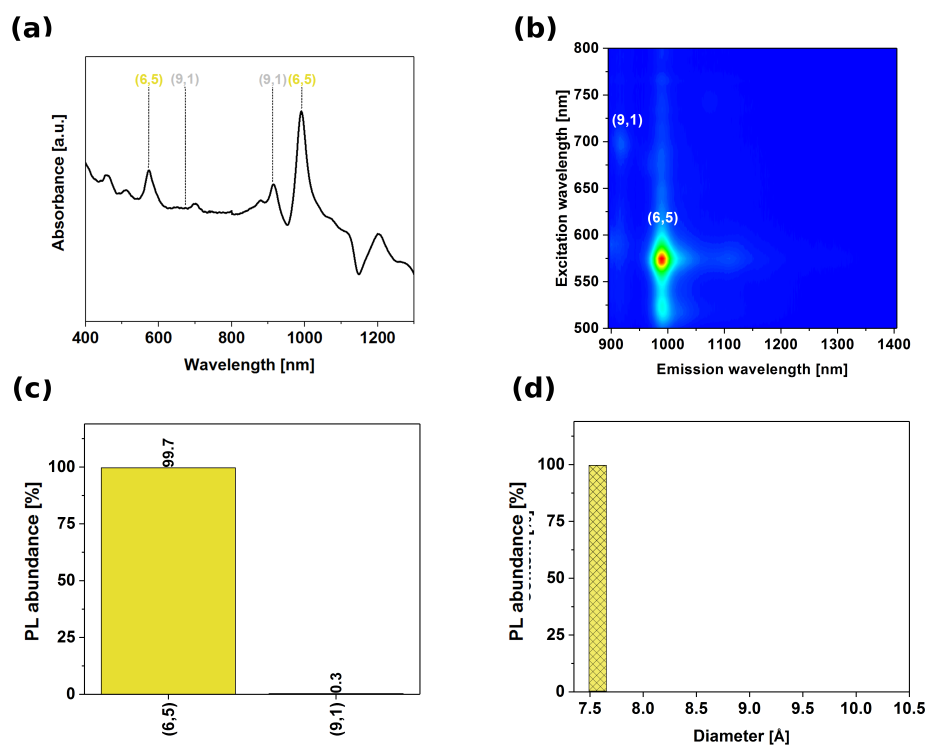


**Figure S7** Characterization of the bottom ATPE phase after combination of 75 µL CNT + 20 µL NH_3_ (a) absorbance spectrum, (b) 2D PL map, (c) Abundance expressed by PL intensity and (d) corresponding diameter distribution.

Table 5. ATPE parameters

| **Compound** | **Aq. concentration [%]** | **Volume [µL]** |
| --- | --- | --- |
| DEX | 20 | 450 |
| PEG | 50 | 180 |
| SC | 10 | 120 |
| SDS | 10 | 60 |
| CNTs | 2% SC, 1 mg/mL | 75 |
| NH_3_ (aq.) | 25 | 20 |
| H_2_O | 100 | 625 |


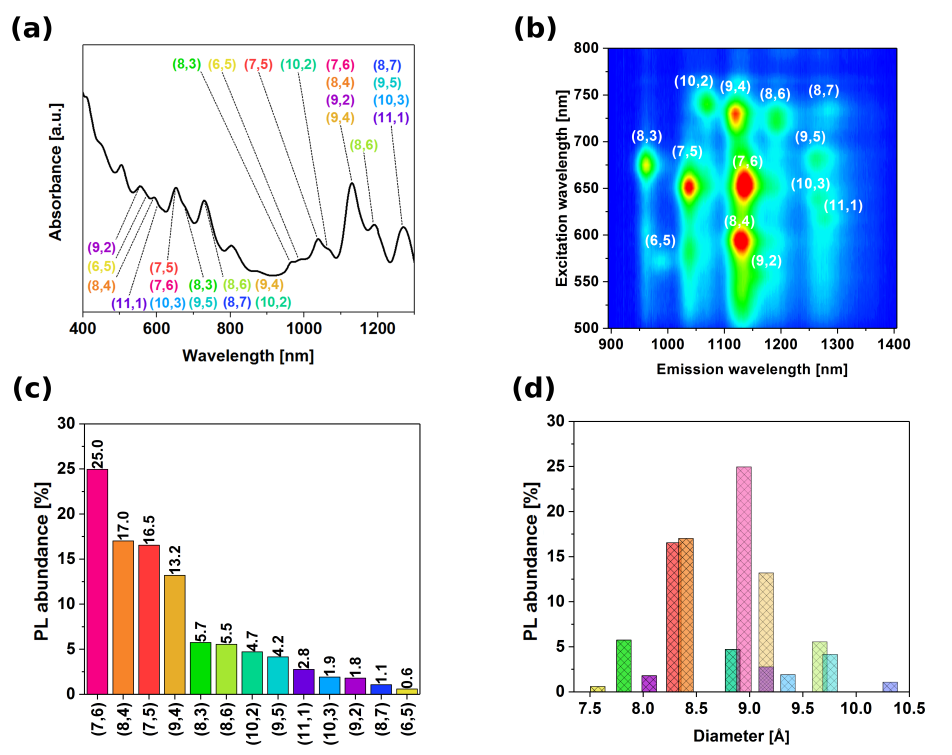


**Figure S8** Characterization of the top ATPE phase after combination of 75 µL CNT + 20 µL NH_3_ (a) absorbance spectrum, (b) 2D PL map, (c) Abundance expressed by PL intensity and (d) corresponding diameter distribution.

Table 6. ATPE parameters

| **Compound** | **Aq. concentration [%]** | **Volume [µL]** |
| --- | --- | --- |
| DEX | 20 | 450 |
| PEG | 50 | 180 |
| SC | 10 | 120 |
| SDS | 10 | 60 |
| CNTs | 2% SC, 1 mg/mL | 75 |
| NH_3_ (aq.) | 25 | 20 |
| H_2_O | 100 | 625 |

- 1. Effect of D_2_O


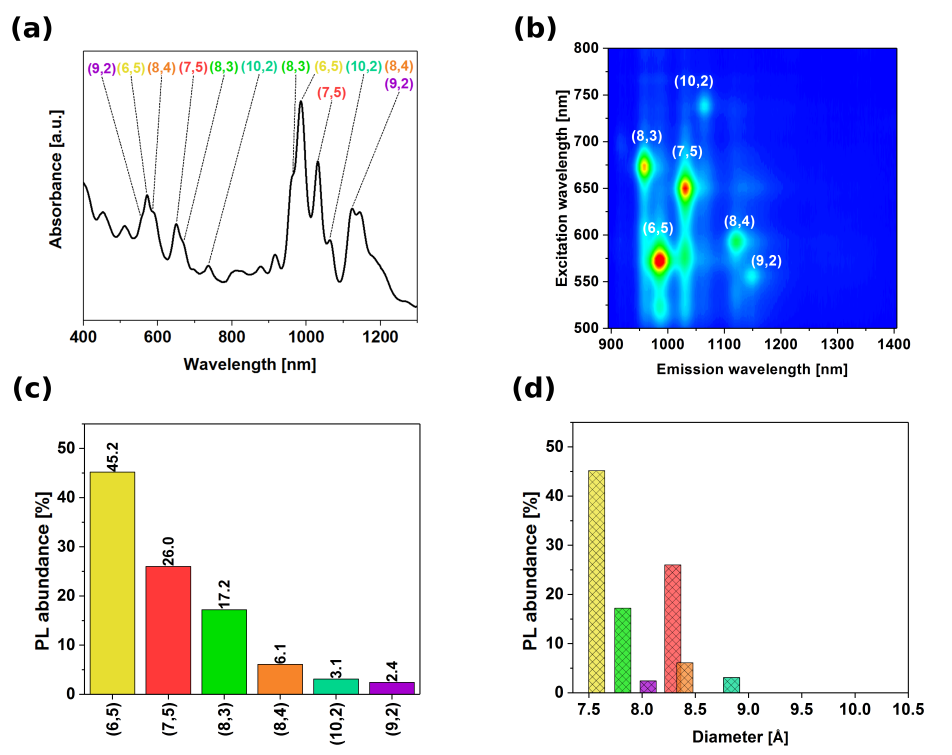


**Figure S9** Characterization of the bottom ATPE phase after combination of 300 µL CNT + 80 µL NH_3_ (a) absorbance spectrum, (b) 2D PL map, (c) Abundance expressed by PL intensity and (d) corresponding diameter distribution. The separation was carried out in D_2_O.

Table 7. ATPE parameters

| **Compound** | **Concentration in D_2_O [%]** | **Volume [µL]** |
| --- | --- | --- |
| DEX | 20 | 450 |
| PEG | 50 | 180 |
| SC | 10 | 120 |
| SDS | 10 | 60 |
| CNTs | 2% SC, 1 mg/mL | 300 |
| NH_3_ (aq.) | 25 | 80 |
| D_2_O | 100 | 340 |


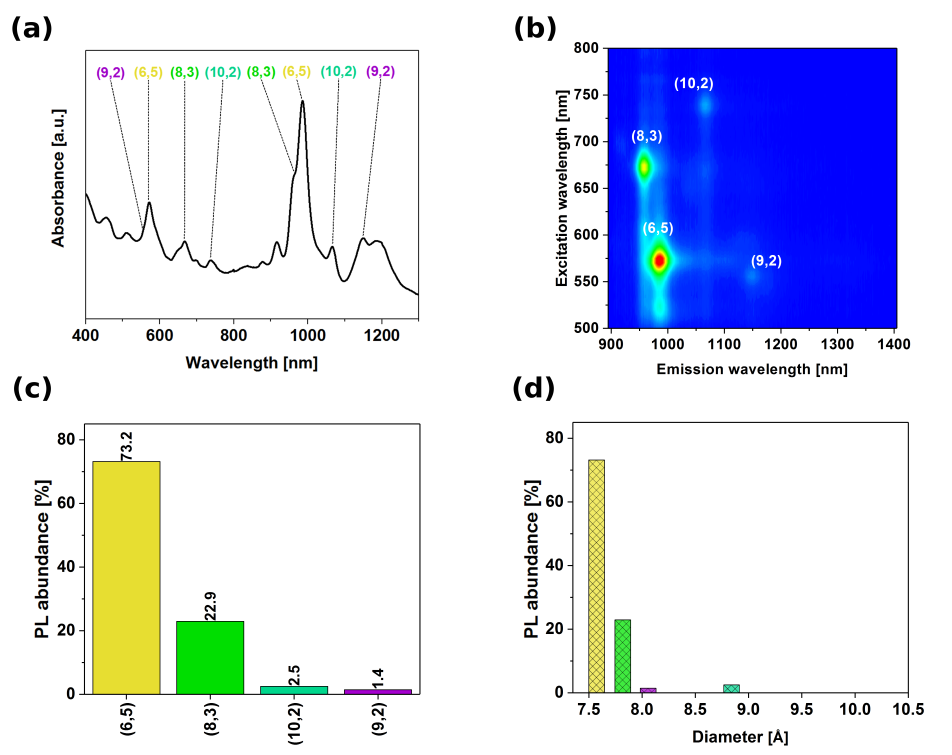


**Figure S10** Characterization of the bottom ATPE phase after combination of 150 µL CNT + 40 µL NH_3_ (a) absorbance spectrum, (b) 2D PL map, (c) Abundance expressed by PL intensity and (d) corresponding diameter distribution. The separation was carried out in D_2_O.

Table 8. ATPE parameters

| **Compound** | **Concentration in D_2_O [%]** | **Volume [µL]** |
| --- | --- | --- |
| DEX | 20 | 450 |
| PEG | 50 | 180 |
| SC | 10 | 120 |
| SDS | 10 | 60 |
| CNTs | 2% SC, 1 mg/mL | 150 |
| NH_3_ (aq.) | 25 | 40 |
| D_2_O | 100 | 530 |


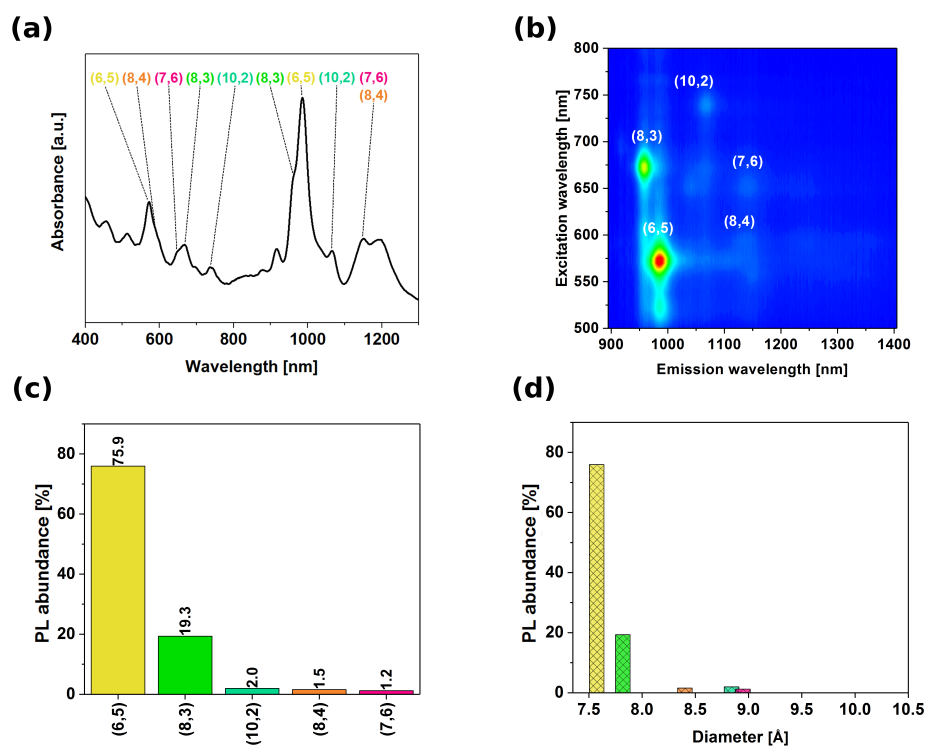


**Figure S11** Characterization of the bottom ATPE phase after combination of 150 µL CNT + 40 µL N_2_H_4_ (a) absorbance spectrum, (b) 2D PL map, (c) Abundance expressed by PL intensity and (d) corresponding diameter distribution. The separation was carried out in D_2_O.

Table 9. ATPE parameters

| **Compound** | **Concentration in D_2_O [%]** | **Volume [µL]** |
| --- | --- | --- |
| DEX | 20 | 450 |
| PEG | 50 | 180 |
| SC | 10 | 120 |
| SDS | 10 | 60 |
| CNTs | 2% SC, 1 mg/mL | 150 |
| N_2_H_4_ (aq.) | 25 | 40 |
| D_2_O | 100 | 530 |


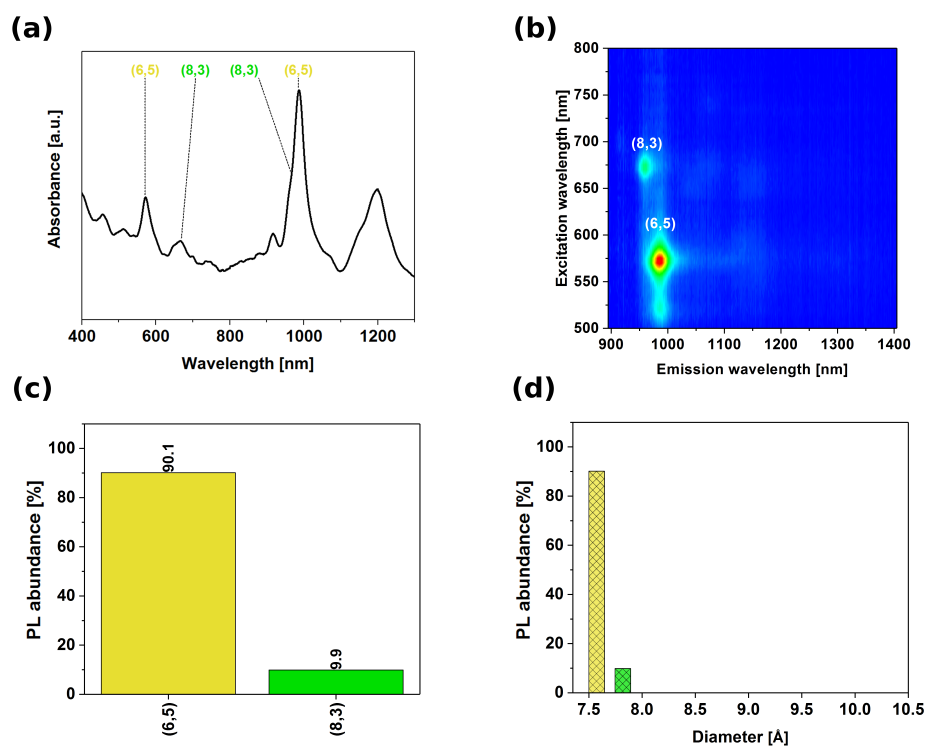


**Figure S12** Characterization of the bottom ATPE phase after combination of 75 µL CNT + 20 µL N_2_H_4_ (a) absorbance spectrum, (b) 2D PL map, (c) Abundance expressed by PL intensity and (d) corresponding diameter distribution. The separation was carried out in D_2_O.

Table 10. ATPE parameters

| **Compound** | **Concentration in D_2_O [%]** | **Volume [µL]** |
| --- | --- | --- |
| DEX | 20 | 450 |
| PEG | 50 | 180 |
| SC | 10 | 120 |
| SDS | 10 | 60 |
| CNTs | 2% SC, 1 mg/mL | 75 |
| N_2_H_4_ (aq.) | 25 | 20 |
| D_2_O | 100 | 625 |

- 1. (8,3) CNTs extraction attempts


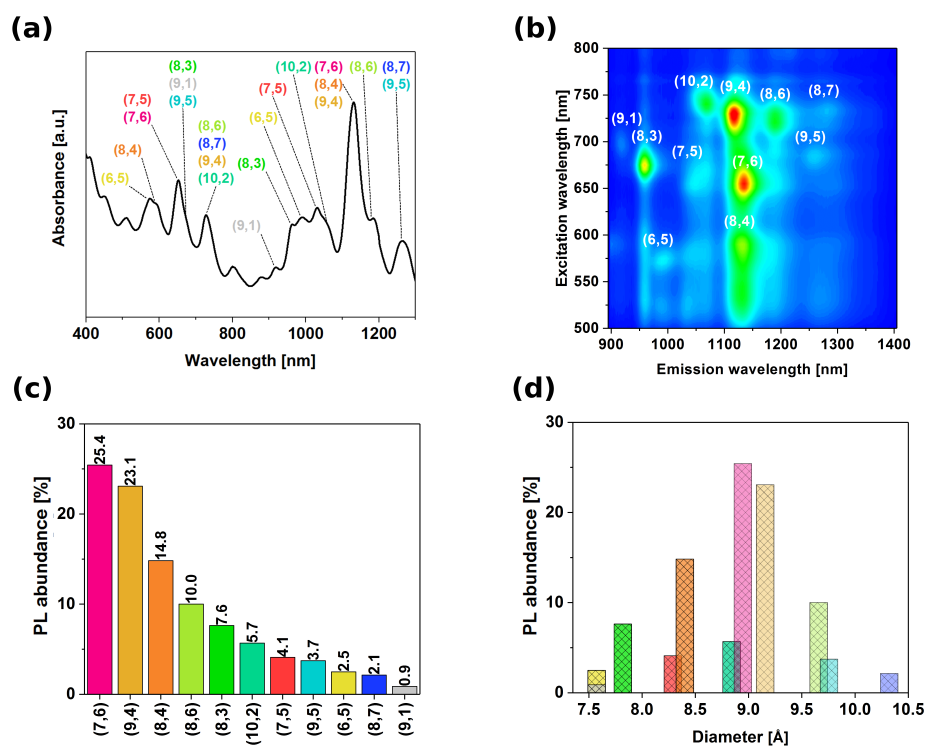


**Figure S13** Characterization of the bottom ATPE phase after combination of 300 µL CNT + 80 µL N_2_H_4_ (a) absorbance spectrum, (b) 2D PL map, (c) Abundance expressed by PL intensity and (d) corresponding diameter distribution.

Table 11. ATPE parameters

| **Compound** | **Aq. concentration [%]** | **Volume [µL]** |
| --- | --- | --- |
| DEX | 20 | 450 |
| PEG | 50 | 180 |
| SC | 10 | 120 |
| SDS | 10 | 60 |
| CNTs | 2% SC, 1 mg/mL | 300 |
| N_2_H_4_ (aq.) | 25 | 80 |
| H_2_O | 100 | 340 |


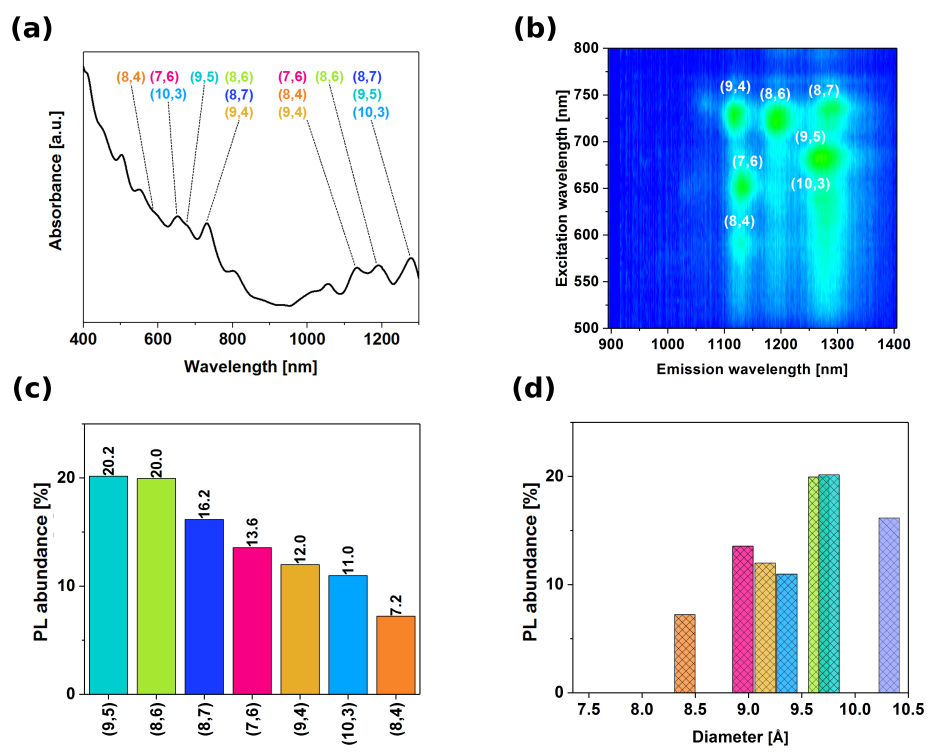


**Figure S14** Characterization of the top ATPE phase after combination of 300 µL CNT + 80 µL N_2_H_4_ (a) absorbance spectrum, (b) 2D PL map, (c) Abundance expressed by PL intensity and (d) corresponding diameter distribution.

Table 12. ATPE parameters

| **Compound** | **Aq. concentration [%]** | **Volume [µL]** |
| --- | --- | --- |
| DEX | 20 | 450 |
| PEG | 50 | 180 |
| SC | 10 | 120 |
| SDS | 10 | 60 |
| CNTs | 2% SC, 1 mg/mL | 300 |
| N_2_H_4_ (aq.) | 25 | 80 |
| H_2_O | 100 | 340 |


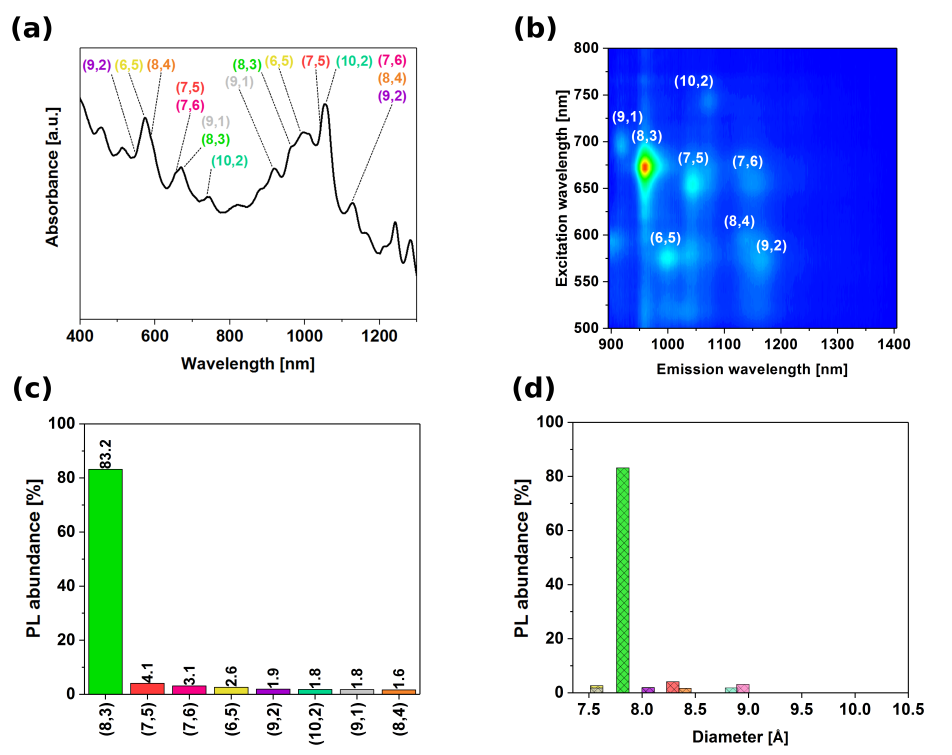


**Figure S15** Characterization of the bottom ATPE phase after combination of 150 µL CNT + 200 µL N_2_H_4_ (a) absorbance spectrum, (b) 2D PL map, (c) Abundance expressed by PL intensity and (d) corresponding diameter distribution.

Table 13. ATPE parameters

| **Compound** | **Aq. concentration [%]** | **Volume [µL]** |
| --- | --- | --- |
| DEX | 20 | 450 |
| PEG | 50 | 180 |
| SC | 10 | 120 |
| SDS | 10 | 60 |
| CNTs | 2% SC, 1 mg/mL | 150 |
| N_2_H_4_ (aq.) | 25 | 200 |
| H_2_O | 100 | 370 |


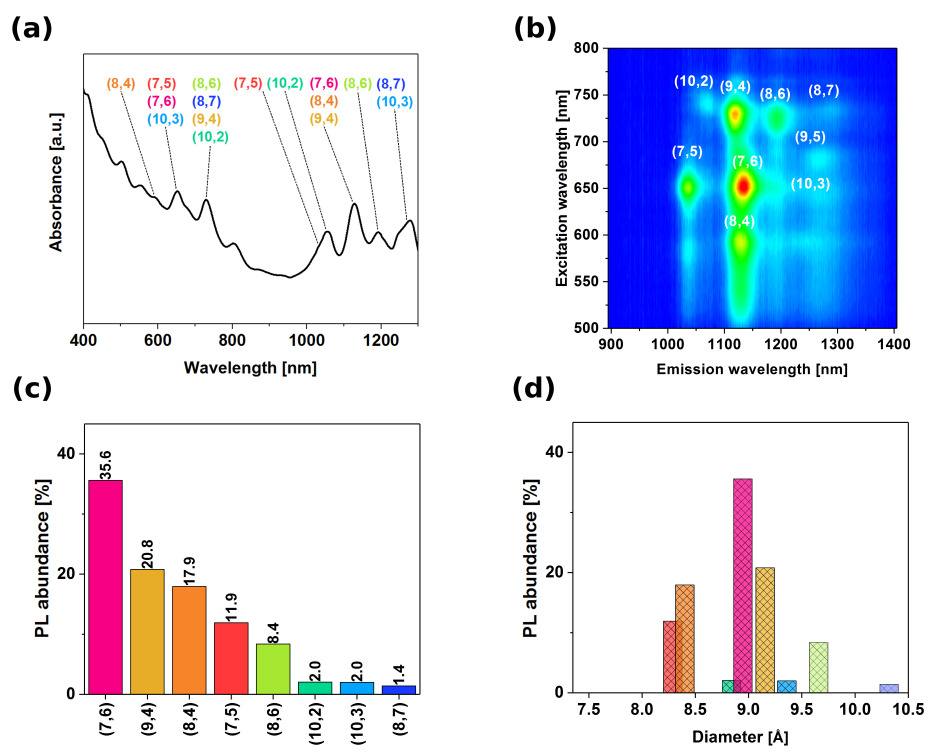


**Figure S16** Characterization of the top ATPE phase after combination of 150 µL CNT + 200 µL N_2_H_4_ (a) absorbance spectrum, (b) 2D PL map, (c) Abundance expressed by PL intensity and (d) corresponding diameter distribution.

Table 14. ATPE parameters

| **Compound** | **Aq. concentration [%]** | **Volume [µL]** |
| --- | --- | --- |
| DEX | 20 | 450 |
| PEG | 50 | 180 |
| SC | 10 | 120 |
| SDS | 10 | 60 |
| CNTs | 2% SC, 1 mg/mL | 150 |
| N_2_H_4_ (aq.) | 25 | 200 |
| H_2_O | 100 | 370 |

- 1. PL from doped-site


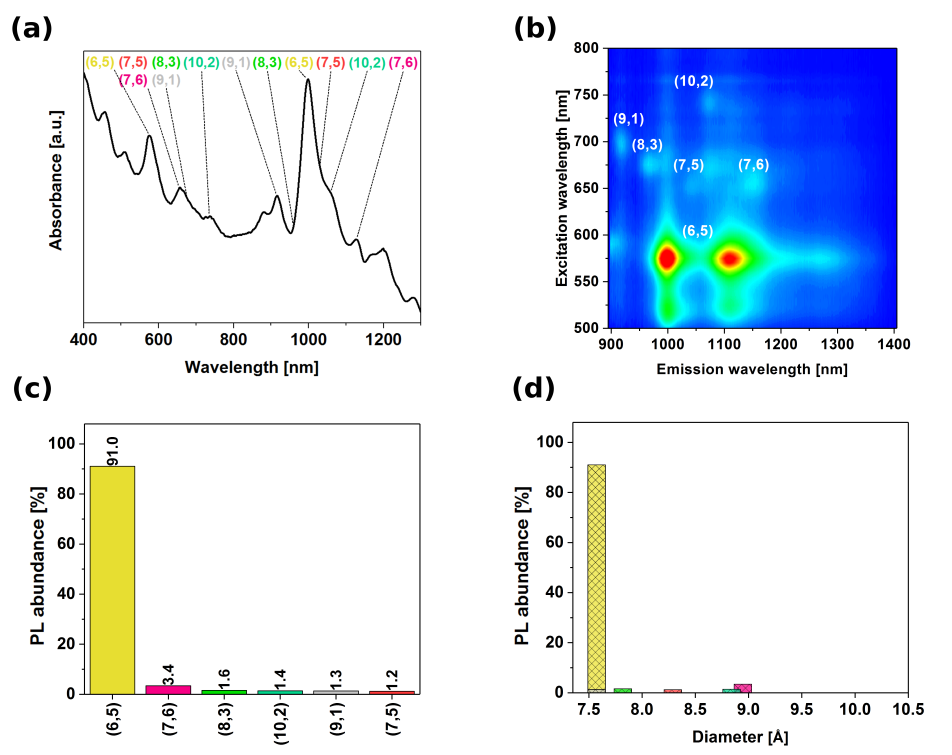


**Figure S17** Characterization of the bottom ATPE phase after combination of 75 µL CNT + 20 µL N_2_H_4_ (a) absorbance spectrum, (b) 2D PL map, (c) Abundance expressed by PL intensity and (d) corresponding diameter distribution.

Table 15. ATPE parameters

| **Compound** | **Aq. concentration [%]** | **Volume [µL]** |
| --- | --- | --- |
| DEX | 20 | 450 |
| PEG | 50 | 180 |
| SC | 10 | 120 |
| SDS | 10 | 60 |
| CNTs | 2% SC, 1 mg/mL | 75 |
| N_2_H_4_ (aq.) | 25 | 20 |
| H_2_O | 100 | 625 |


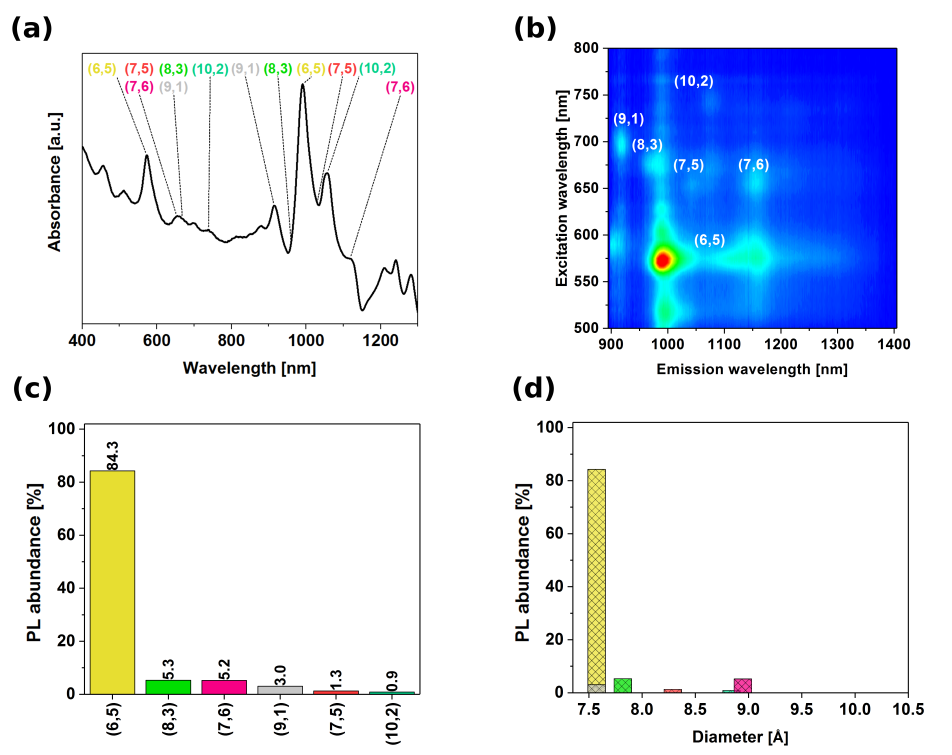


**Figure S18** Characterization of the bottom ATPE phase after combination of 75 µL CNT + 100 µL N_2_H_4_ (a) absorbance spectrum, (b) 2D PL map, (c) Abundance expressed by PL intensity and (d) corresponding diameter distribution.

Table 16. ATPE parameters

| **Compound** | **Aq. concentration [%]** | **Volume [µL]** |
| --- | --- | --- |
| DEX | 20 | 450 |
| PEG | 50 | 180 |
| SC | 10 | 120 |
| SDS | 10 | 60 |
| CNTs | 2% SC, 1 mg/mL | 75 |
| N_2_H_4_ (aq.) | 25 | 100 |
| H_2_O | 100 | 545 |


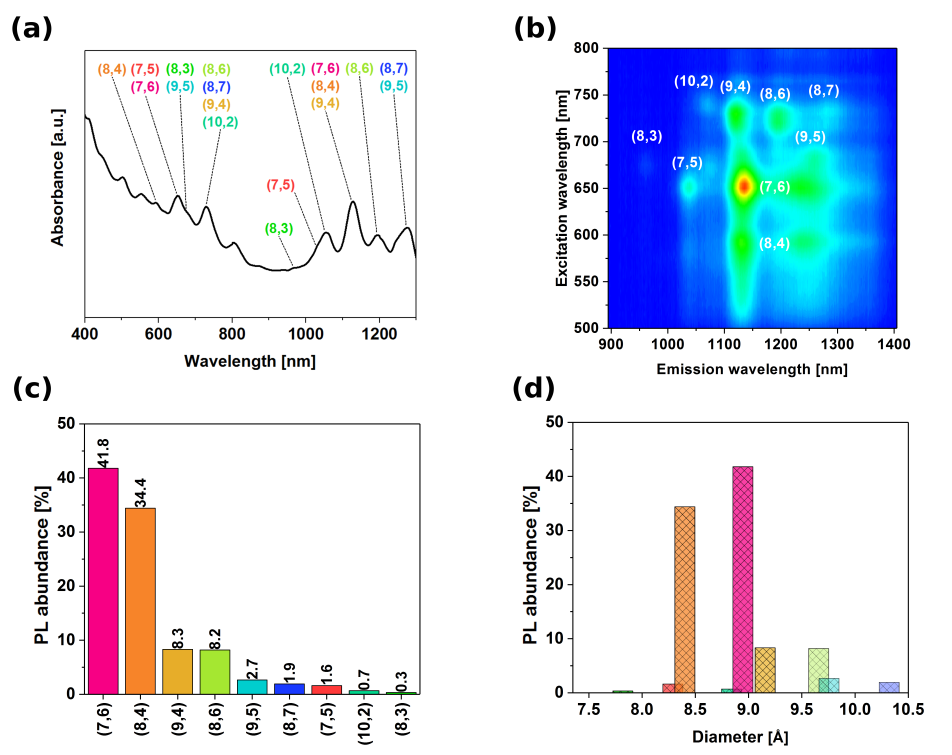


**Figure S19** Characterization of the top ATPE phase after combination of 75 µL CNT + 100 µL N_2_H_4_ (a) absorbance spectrum, (b) 2D PL map, (c) Abundance expressed by PL intensity and (d) corresponding diameter distribution.

Table 17. ATPE parameters

| **Compound** | **Aq. concentration [%]** | **Volume [µL]** |
| --- | --- | --- |
| DEX | 20 | 450 |
| PEG | 50 | 180 |
| SC | 10 | 120 |
| SDS | 10 | 60 |
| CNTs | 2% SC, 1 mg/mL | 75 |
| N_2_H_4_ (aq.) | 25 | 100 |
| H_2_O | 100 | 545 |

1. Corresponding author. Tel/Fax: +48 32 2372958. E-mail address: dawid.janas@polsl.pl (D. Janas). [↑](#footnote-ref-1)
